# Global analysis of the effect of local climate on the hatchling output of leatherback turtles

**DOI:** 10.1038/srep16789

**Published:** 2015-11-17

**Authors:** Pilar Santidrián Tomillo, Vincent S. Saba, Claudia D. Lombard, Jennifer M. Valiulis, Nathan J. Robinson, Frank V. Paladino, James R. Spotila, Carlos Fernández, Marga L. Rivas, Jenny Tucek, Ronel Nel, Daniel Oro

**Affiliations:** 1 Population Ecology Group, Institut Mediterrani d’ Estudis Avançats, IMEDEA (CSIC-UIB), Miquel Marquès, 21, 07190, Esporles, Mallorca, Spain; 2 National Oceanic and Atmospheric Administration, National Marine Fisheries Service, Northeast Fisheries Science Center, c/o Geophysical Fluid Dynamics Laboratory, 201 Forrestal Road, Princeton University Forrestal Campus, Princeton, New Jersey, USA; 3 U.S. Fishing and Wildlife Service, USA; 4 Geographic Consulting, St. Croix, U.S. Virgin Islands, USA; 5 Department of Biology, Indiana-Purdue University, Fort Wayne, Indiana, USA; 6 Department of Biodiversity, Earth and Environmental Science, Drexel University, Philadelphia, Pennsylvania, USA; 7 Endangered Wildlife Trust, Avda. 11, San José, Costa Rica; 8 Universidad de Granada, Campus Fuentenueva s/n, Granada, Spain; 9 Department of Zoology, Nelson Mandela Metropolitan University, Port Elizabeth, South Africa

## Abstract

The most recent climate change projections show a global increase in temperatures along with precipitation changes throughout the 21^st^ century. However, regional projections do not always match global projections and species with global distributions may exhibit varying regional susceptibility to climate change. Here we show the effect of local climatic conditions on the hatchling output of leatherback turtles (*Dermochelys coriacea*) at four nesting sites encompassing the Pacific, Atlantic and Indian Oceans. We found a heterogeneous effect of climate. Hatchling output increased with long-term precipitation in areas with dry climatic conditions (Playa Grande, Pacific Ocean and Sandy Point, Caribbean Sea), but the effect varied in areas where precipitation was high (Pacuare, Caribbean Sea) and was not detected at the temperate site (Maputaland, Indian Ocean). High air temperature reduced hatchling output only at the area experiencing seasonal droughts (Playa Grande). Climatic projections showed a drastic increase in air temperature and a mild decrease in precipitation at all sites by 2100. The most unfavorable conditions were projected for Sandy Point where hatching success has already declined over time along with precipitation levels. The heterogeneous effect of climate may lead to local extinctions of leatherback turtles in some areas but survival in others by 2100.

While global air temperatures are projected to increase due to anthropogenically-induced climate change, these projections vary regionally^1^. Similar variation is also expected in precipitation patterns, such that mean rainfall/snowfall is projected to increase in some areas but decline in others. As climate may vary over a species range, the effects of climate change may be spatially heterogeneous resulting in higher extinction risk of a species in some areas than in others^2^.

Sea turtles have global distributions although nesting beaches are limited to tropical or subtropical areas of the Pacific, Atlantic and Indian Ocean basins. Egg development and hatchling emergence are affected by prevailing climatic conditions^3^,^4^. Temperatures at nest depth are usually highly correlated to air temperature^5^,^6^,^7^ and affected by precipitation^3^. High temperatures in the nest increase (1) mortality of eggs and hatchlings^8^,^9^,^10^ and (2) production of female hatchlings^11^, often resulting in female biased primary sex ratios^12^,^13^,^14^. Precipitation levels also directly affect sea turtle clutches. For instance, high levels of precipitation can benefit egg development and hatchling emergence^4^ and cause a cooling effect in the nest environment^3^. Nevertheless, excessive precipitation can be detrimental to sea turtle clutches by causing egg suffocation, especially when eggs are developing in poorly drained sand^15^.

Hatchling output of leatherback turtles (*Dermochelys coriacea*) in the eastern Pacific fluctuates with the prevailing climatic conditions driven by El Niño Southern Oscillation (ENSO) in the region. During El Niño years, low levels of precipitation and high air temperatures result in lower hatchling production than during La Niña events, when precipitation levels are high^4^. Because high temperature and low precipitation negatively affect hatchling output, population declines are projected for this area as conditions become unfavorable for egg development and hatchling emergence throughout the 21^st^ century^16^.

Our main objective was to analyze the effect of local climatic conditions on the hatchling output of leatherback turtles in a global manner. We additionally wanted to identify populations that could be more susceptible to climate change by using global climate model projections for each study site through the end of the 21^st^ century. This information is particularly pertinent because the IUCN assessment of leatherback turtles recently changed from critically endangered to vulnerable but included regional assessments^17^. Whereas East Pacific and Southwest Indian subpopulations are still considered critically endangered due to low numbers of nesting females and declining trends, the Northwest Atlantic subpopulations are classified as least concern as populations are stable or increasing. If susceptibility to climate change differs among leatherback populations, future IUCN classifications should consider including this type of analysis in their assessments.

## Results

There were significant differences in mean hatching success (one-way ANOVA, F = 15.269, p < 0.001) and mean emergence rate (F = 26.747, p < 0.001) between sites. The post-hoc test indicated that hatching success was significantly higher at the temperate site (Maputaland, mean ± SD: 0.80 ± 0.06), than at the tropical sites (Playa Grande: 0.46 ± 0.12, p < 0.001; Sandy Point: 0.64 ± 0.09, p < 0.01 and Pacuare: 0.59 ± 0.06, p < 0.01). There were also significant differences in hatching success between Sandy Point and Playa Grande (p < 0.001) and Pacuare and Playa Grande (p < 0.05), but not between the two Caribbean sites (Sandy Point and Pacuare, p = 0.584). Highest emergence rate was also registered for the temperate site (Maputaland: 0.97 ± 0.01), followed by Sandy Point (0.94 ± 0.02), Pacuare (0.91 ± 0.05) and Playa Grande (0.83 ± 0.06). Significant differences in emergence rate were found between Maputaland and Playa Grande (p = 0.001), Maputaland and Sandy Point (p < 0.05), and Playa Grande and Sandy Point (p < 0.05), but not between Pacuare and the other three sites (p = 0.358, p = 0.098, p = 0.0502 for comparisons with Sandy Point, Playa Grande and Maputaland respectively).

### Effect of local climatic conditions on hatchling output

**Figure 1:**
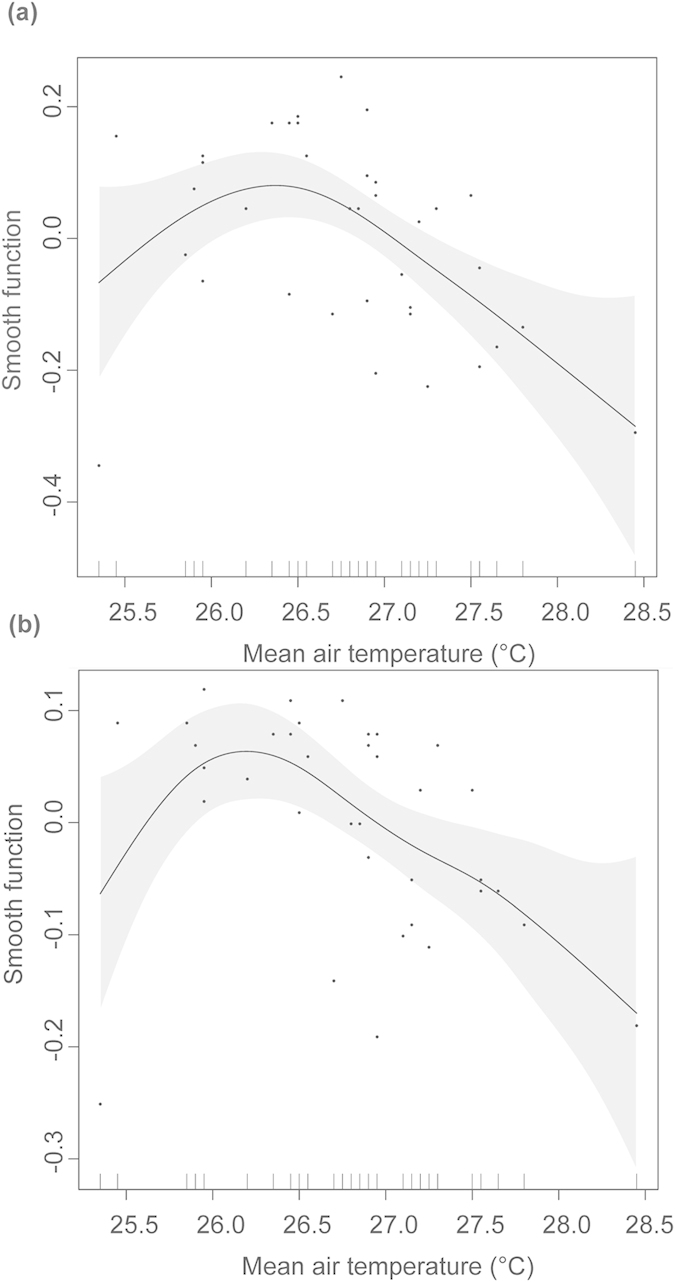
Generalized Additive Model (GAM) fits of air temperature (°C) against hatchling output at Playa Grande. Smooth fits from GAMs show the additive effects of mean air temperature (°C) during the two months of incubation on mean monthly (**a**) hatching success and (**b**) emergence rate of leatherback turtle clutches at Playa Grande. Tick marks on the x-axis represents observed data points and Y-axis represents the smooth function and the values are centered. Gray area corresponds to two standard errors around the main effect.

Overall, local precipitation significantly affected hatching success and emergence rate of leatherback turtle clutches when data from all sites were combined and also separately at Sandy Point, Playa Grande and Pacuare. On the contrary, there was not a general effect of air temperature on hatchling output when we combined data from all sites, and air temperature only had a significant effect on hatching success and emergence rate when analyzed independently for Playa Grande (Fig. 1).

#### Hatching success

The effect of precipitation on hatching success varied among sites. The two GAMs that best resolved the variability in hatching success when we combined data from all sites (lowest AICc) was a function of the rainfall accumulated during the incubation months together with the average air temperature for the same time, as well as the model that included average monthly precipitation and air temperature during the incubation months (supplementary Table S1). The effect of rain was significant in both models (p < 0.001), but not the effect of air temperature (p = 0.118 both models).

**Figure 2:**
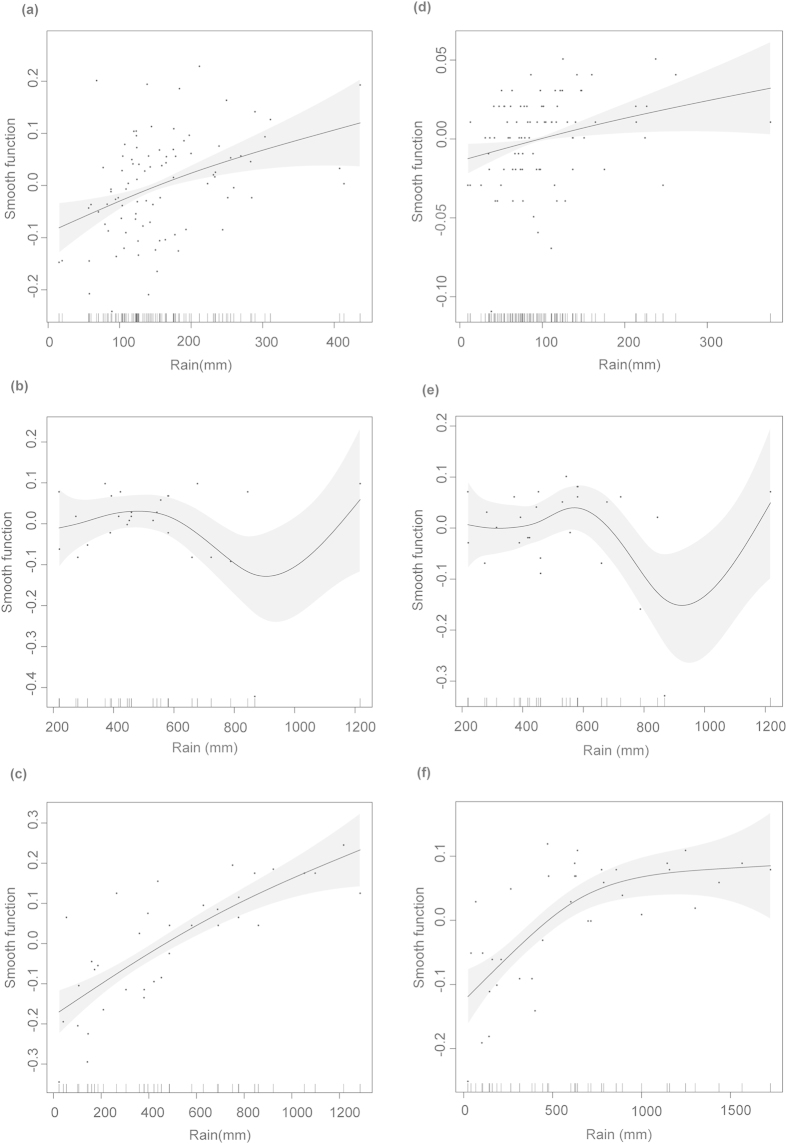
Generalized Additive Model (GAM) fits of local climatic conditions against hatchling output. Smooth fits show the additive effects of significant predictors on mean monthly hatching success and emergence rate. Best predictors on hatching success were precipitation accumulated during **(a)** the month eggs were laid and the two previous months at Sandy Point, **(b)** the month eggs were laid and the month before at Pacuare and **(c)** the two months before eggs were laid at Playa Grande. Emergence rate predictors were precipitation accumulated **(d)** two months before eggs were laid at Sandy Point, **(e)** during the month eggs were laid and the month before at Pacuare and **(f)** in the month eggs were laid and two months before at Playa Grande. Best models were selected based on the minimum Akaike Information Criterion (AICc). Tick marks on the x-axis represents observed data points. Y-axis represents the smooth function and the values are centered. Gray area corresponds to two standard errors around the main effect. See supplementary Fig. S1 online for a representation of hatching success and emergence rate values against precipitation.

At Sandy Point, the model with the lowest AICc was the one including average air temperature and rain accumulated during the month eggs were laid and the two months before. However, the effect of precipitation on hatching success was significant (p < 0.01) but the effect of temperature was not (p = 0.14). Hatching success at Sandy Point increased with precipitation (Fig. 2a). At Playa Grande, the model with the lowest AICc included rain accumulated two months before eggs were laid (p < 0.001). Likewise, hatching success at Playa Grande increased with precipitation (Fig. 2c). At Pacuare, the model with the lowest AICc included rain accumulated during the month eggs were laid and the previous month and although the model explained 31.6% of the deviance, its effect was not significant. However, the model that included rain accumulated two months before eggs were laid had a significant effect (p < 0.05) (supplementary Table S1). At Pacuare, hatching success did not show a clear relationship to precipitation levels (accumulated over 2 months) lower than 600 mm, but declined at precipitation levels between 600 mm to 900 mm with an apparent increase at levels greater than 900 mm (Fig. 2b). None of the predictor variables tested by any model had a significant effect on hatching success at Maputaland.

**Figure 3:**
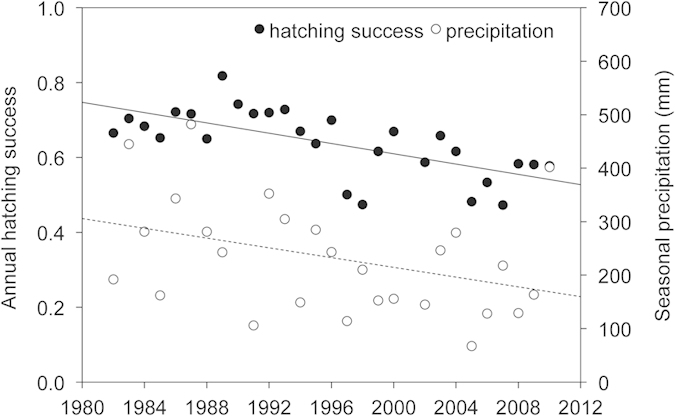
Changes in annual hatching success and seasonal precipitation (mm) over time at Sandy Point. Black and dash lines show the declining trends in hatching success and seasonal precipitation respectively. Seasonal precipitation corresponds to precipitation accumulated over the four months of the nesting season.

At Sandy Point, the amount of seasonal rainfall has decreased over time likely contributing to the decline in hatching success over the same period (Fig. 3). This trend was not evident at the other sites.

#### Emergence rate

When data from all sites were combined, the analyses showed that local precipitation significantly affected emergence rate. The model with the lowest AICc included average precipitation during the months of incubation. The model that included the effect of rain accumulated during the two months of incubation had a very similar AICc (supplementary Table S2, p < 0.001 in both cases). At Sandy Point, the best model included rain accumulated in the two months before eggs were laid; however, it only explained 8.4% of the deviance (p < 0.05, Fig. 2d). At Playa Grande, the best model included rain accumulated during the month eggs were laid and two months before (p < 0.001, Fig. 2f). This model explained 55.8% of the deviance. The model that included air temperature and rain accumulated over the same period of time explained 77.7% of deviance and had a significant effect (p < 0.05) but its AICc was not lower than that of the previous model. At Pacuare, the best model included rain accumulated during the month eggs were laid and the month before. However its effect was not significant although it was close to being significant (p = 0.07, Fig. 2e). None of the predictor variables tested by any model had a significant effect on the emergence rate at Maputaland.

### Climate projections for leatherback nesting sites through the 21^st^ century

**Figure 4:**
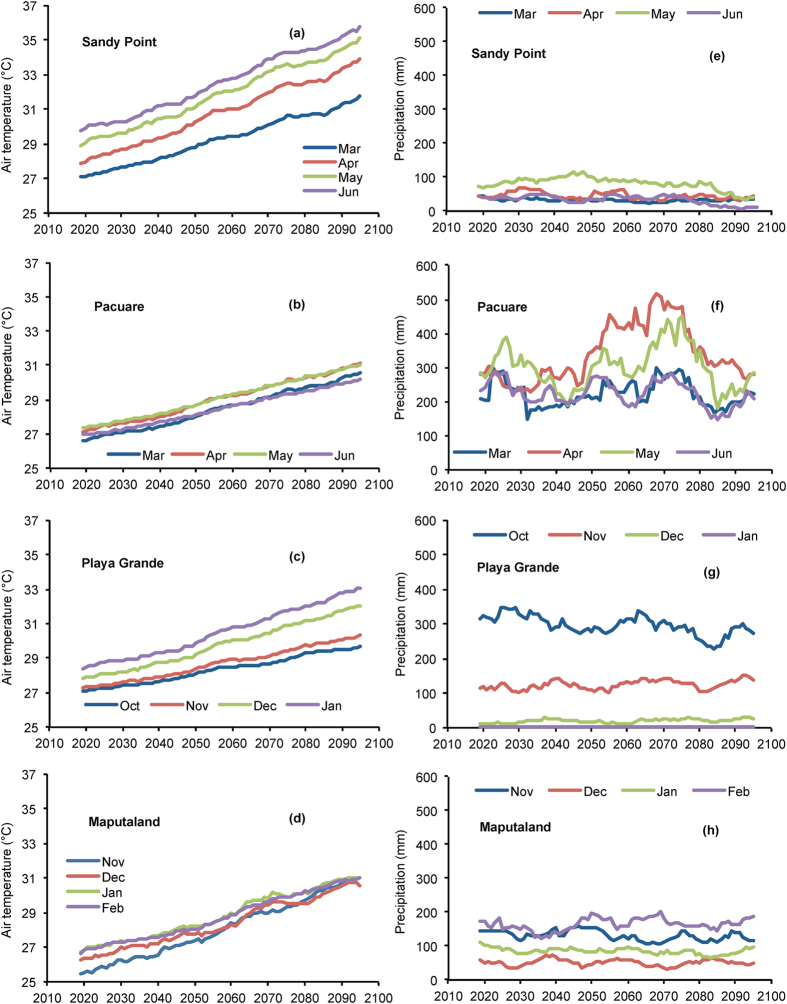
Projections of local climatic conditions throughout the 21^st^ century. Projections of air temperature (°C) are for **(a)** Sandy Point, **(b)** Pacuare, **(c)** Playa Grande and **(d)** Maputaland and projections of accumulated precipitation (mm) for **(e)** Sandy Point, **(f)** Pacuare**, (g)** Playa Grande and **(h)** Maputaland. Projections correspond to months of the nesting season for each nesting site and are presented as 10 year moving averages.

Global climate model projections throughout the 21^st^ century showed an increase in air temperature at all sites (Fig. 4, Table 1), but the magnitude of change varied among sites. The greatest warming was projected for Sandy Point (+5.4 °C) and Maputaland (+5 °C) followed by Pacuare (+3.9 °C) and Playa Grande (+3.5 °C). Highest mean seasonal temperatures by 2100 were projected for Sandy Point (33.4 °C) and were about 2.5 °C warmer than at the other sites (Playa Grande: 30.9 °C, Pacuare: 30.8 °C and Maputaland: 31.0 °C). The rate of warming also varied among months within the nesting season at Playa Grande and Sandy Point such that warming was milder in some months than in others. Both at Playa Grande and at Sandy Point, the warming was greatest towards the end of the season (+5.0 °C in January at Playa Grande and +7.1 °C in May at Sandy Point) than in the early months (+2.5 °C in November at Playa Grande and +4.2 °C in March at Sandy Point). However, Pacuare and Maputaland showed a similar increase among months of the nesting season (Fig. 4).

Only at Maputaland, projections showed that air temperature and precipitation during some months (July-October) would be similar to contemporary nesting season conditions (Table 1).

Generally, projections from the global climate models showed a slight trend for drier conditions by 2100 (Table 1, Fig. 4). However, the change was subtle and with a slight increase in rainfall in some months (Table 1). Only the projections for Pacuare showed higher variability with an increase in precipitation towards the middle of the 21^st^ century and a decrease towards the end (Fig. 4f).

## Discussion

Rainfall can flush salt from the beach, increase water content of sand, decrease drying front above incubating eggs^18^ and have a cooling effect on nest temperatures^3^, but high levels of precipitation can also result in egg suffocation and clutch failure^16^. Sea turtle eggs need moisture for successful development^18^,^19^, but moisture levels that are too high or too low can reduce hatching success^20^.

Precipitation was the main climatic driver of leatherback hatchling output around the World but its effects varied at different sites. We found a positive effect of rainfall on the hatchling output (Fig. 2) in areas where clutches experience climatic stress due to either extended dry seasons (Playa Grande) or low average rainfall (Sandy Point). This suggests that it is the lack of rain in these areas what reduces developmental success and hatchling emergence. Additionally, precipitation accumulated over time (2–3 months) had the strongest effect on hatchling output in the tropical areas, suggesting a long-lasting effect of rainfall on the nest environment.

There is a growing interest in developing climate mitigation strategies such as shading and nest irrigation in preparation for unfavorable climate change scenarios^21^,^22^. Our results imply that climate-mitigation strategies on the nesting beaches should be site-specific and consider the effect of rainfall on the nest environment. Mitigation studies have generally focused on reducing nest temperatures to avoid female-biased primary sex ratios and reduce embryo mortality^23^. However, recent studies show that (1) operational sex ratios are not as female-biased as previously thought because males can reproduce more often than females^24^, (2) naturally changing sex ratios may be adaptive and female-biased primary sex ratios advantageous under warming conditions^25^ and (3) sea turtle embryos of some species may be more resilient to high temperatures than previously thought^26^. Additionally, similar climatic conditions may also affect the nest environment in different ways depending on nest position within the beach and sand albedo^5^.

Our findings also suggest that long-term watering may create a suitable nest environment, which constitutes a simple mitigation action that could be done before clutches are laid. This would avoid direct watering of clutches and alteration of sex ratios. However, we still caution researchers against implementing such strategies unless, hatchling output reaches a critically low point – a value that still needs to be determined.

At the rainiest site, the effect of precipitation on hatching success varied. Hatching success first declined as precipitation increased but then increased at the highest precipitation levels. The decline in hatching success with rain could result from direct soil saturation or rise of the water table level that displaced air in between sand particles leading to suffocation. However, the increase in hatching success at the highest precipitation levels is puzzling, although it could be a spurious result. Indeed, this increase corresponded to a single event of unusually high precipitation levels registered in 2009, when extreme erosion events removed large sectors of the beach and a high percentage of clutches were relocated from high-risk areas (>70%). *In situ* clutches that were not relocated incubated in the most favorable areas, which could explain the high mean hatching success.

Surprisingly, the effect of air temperature in most cases was not significant despite being included as a predictor in some of the best models. High temperature in the nest is known to reduce hatching success of sea turtle eggs and emergence rate of hatchlings^8^,^9^,^27^,^28^,^29^. However, we found that air temperatures only significantly influenced hatchling output at Playa Grande - the site experiencing seasonal droughts. Because of the extremely dry conditions registered at the end of the season in this area, nest temperatures are likely higher than those experienced at locations that receive rain during incubation. Sand temperatures can increase rapidly without rain in areas with distinctive wet/dry seasons^8^,^27^. Thus, at these locations prolonged lack of rain likely cause hydric and thermal stresses on developing eggs and emerging hatchlings, reducing overall hatchling output. In addition to the effect of the rainfall itself, the presence of rainfall requires the presence of cloud cover and cloud cover causes a decrease in solar radiation by as much as 2/3^30^. That decrease causes a reduction in solar heating and therefore causes a cooling of the sand and reduces nest temperatures.

Air temperatures are projected to increase at all sites throughout the 21^st^ century and precipitation is projected to show a mild decrease. However, some areas are expected to get warmer, warm at a higher rate and experience greater intra-annual variability in climatic conditions than others. The highest warming rate, highest absolute temperature and lowest precipitation levels were projected for Sandy Point, an area where hatching success is already declining due to a drying trend (Fig. 3). Further increases in dryness and air temperatures are expected to worsen the hydric and thermal stresses of clutches, likely compromising hatchling output by the end of the 21^st^ century in this area.

Air temperatures during the nesting season may become too high at all sites by 2100, but temperate areas may be more resilient to climate change than tropical regions because of their higher seasonal variability. We did not find an effect of local climate on hatchling output at the temperate site. Conditions at this site were considerably cooler than at the tropical locations and clutches may not suffer from droughts, excessive rainfall or high nest temperatures under current climatic regimes, resulting in the higher hatchling output. Recent studies suggest that ectothermic organisms may be less vulnerable to climate change in temperate areas that in tropical ones^31^. Temperate areas are characterized by a greater daily fluctuation in temperature compared to tropical areas and night temperatures could help to cool the nests under warmer conditions. Additionally, months in the year that currently register temperatures too low for egg development may provide suitable conditions by the end of the 21^st^ century as the climate warms up.

A change in the phenology to nesting a few months earlier at the temperate site, would allow egg clutches to experience similar climatic conditions than those currently encountered during the nesting season. Changes in phenology have been observed in other sea turtles in response to changes in Sea Surface Temperature (SST) with a tendency to nesting earlier^32^,^33^. However, recent studies indicate that patterns of nesting phenology in leatherback turtles do not appear to be strongly affected by oceanographic conditions, suggesting that these animals may not substantially change the timing of the nesting season in response to climate change^34^. Additionally, air temperatures in the tropical sites during the coolest month would still be several degrees warmer than current mean seasonal temperatures (Table 1). Thus, changes in phenology may be inefficient for leatherback turtles that nest in tropical sites.

Another possible phonological response is the shortening of the nesting season. For instance, at Playa Grande air temperatures during two months of the year (Oct-Nov) by the end of the 21^st^ century, could be similar to those currently experience at the end of the dry season. Rain during these months could allow development by cooling the nests. However, the large difference in projected temperatures between these months potentially suitable for development, and the preceding and following months (Table 1) may act as thermal barriers shrinking the nesting season. Such shortening of the nesting season has been observed in loggerhead turtles in Florida in response to increased SST^35^. Short nesting seasons are typical of temperate areas such as the Mediterranean Basin^36^,^37^, where there is a large inter-monthly variability in climatic conditions.

Climate change can further affect sea turtle nesting beaches through sea-level rise and increased frequency of storms/hurricanes and large portions of nesting beaches could become inundated^38^,^39^. Sea-level rise may further intensify the effect of climate change in some leatherback nesting beaches, depending on the regional projections and the physical characteristics of the beach (e.g. elevation and slope).

Although we found that air temperature did not significantly affect hatchling output at most locations, clutches will likely be adversely affected by the ~3.5 °C to 5.5 °C increases in air temperatures by the end of the 21^st^ century because of the observed effects of nest temperature on hatchling output^27^,^29^ and of air temperature found at the driest site in this study. As we start understanding the effects that climate change can have on sea turtle populations, we should start incorporating this impact into species assessments. Local variation in climatic conditions means that differing nesting sites will respond differently under conditions of climate change. It is therefore essential that we focus conservation efforts to protect nesting beaches over a wide-range of distant sites.

## Methods

### Nesting sites and local climate

**Figure 5:**
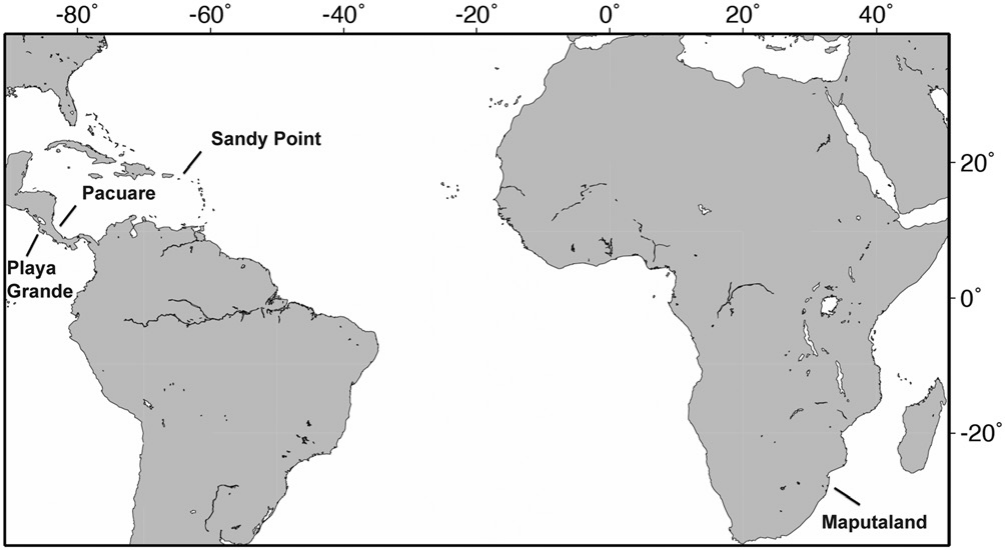
Nesting sites of leatherback turtles (*Dermochelys coriacea*) included in the study. Locations were in the eastern Pacific Ocean (Playa Grande, Costa Rica), in the Atlantic Basin/Caribbean Sea (Pacuare Reserve, Costa Rica and Sandy Point, St. Croix, U.S. Virgin Island) and in the Indian Ocean (Maputaland, South Africa). We generated the map using the Maptool software from SEATURTLE.ORG Maptool. 2002.

We included data from one location in the eastern Pacific Ocean (Playa Grande, Costa Rica, 10°20 N, 85°51 W), two locations in the Atlantic Basin/Caribbean Sea (Pacuare, Costa Rica, 10°12N, 83°15 W and Sandy Point, St. Croix, US Virgin Islands, 17°41 N, 64°54 W) and one location in the Indian Ocean (Maputaland, South Africa, 27°00 S, 32°51 E) (Fig. 5). The populations that nest at Playa Grande and Maputaland are currently considered critically endangered and those that nest at Sandy Point and Pacuare are classified as ‘least concern’^17^ (Table 2). Sites were selected because of data availability and local climatic conditions.

**Figure 6:**
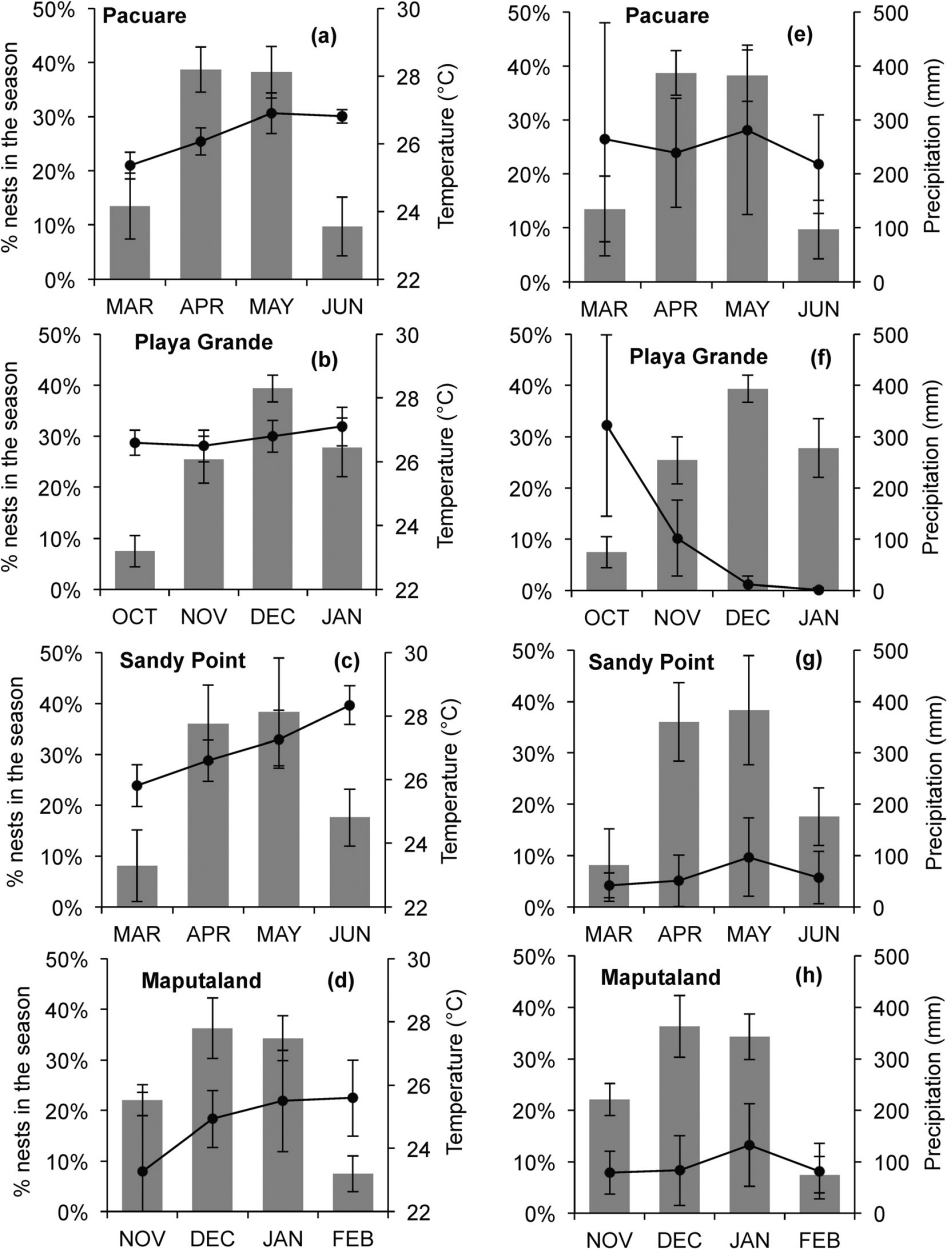
Local climatic conditions and distribution of the nesting season. Mean (±SD) percentage of clutches laid per month (columns) at Playa Grande, Pacuare, Sandy Point and Maputaland versus **(a–d)** mean (±SD) air temperature (°C) and **(e–h)** precipitation (mm) accumulated (black lines) during the same time for each site.

For the purpose of the study, we considered the nesting season to be the time when 80–90% of clutches were laid in a year. Nesting seasons lasted for four months: October-January at Playa Grande, March- June at Pacuare and Sandy Point and November-February at Maputaland (Fig. 6). This classification was based on the months when eggs were laid, since development still continued on those clutches laid on the last month of the season (mean incubation period: ca. 60 days). At Sandy Point and Maputaland, levels of precipitation are relatively low yet consistent throughout the nesting season (~100 mm per month or less). At Pacuare, levels of precipitation are relatively high throughout the season (monthly mean 200–300 mm). At Playa Grande, precipitation is high before and during the first month of the nesting season (October), but rainfall is negligible for 4–5 months starting in the middle of the nesting season (December) (Fig. 6f). Playa Grande is also located in an area that is highly influenced by ENSO and as a result, precipitation and air temperature are highly variable among years^4^. Maputaland, is located within a subtropical/temperate region and air temperature during the nesting season is ~2 °C cooler than at the other sites (Fig. 6).

### Hatchling output, climate data and climate projections

We analyzed 30 years of data on hatchling output from Sandy Point (1982 to 2010; n = 104 months and 3342 clutches), 9 years from Playa Grande (2004–2005 to 2012–2013, n = 36 months and 1125 clutches), 7 years from Pacuare (2008-2014, n = 27 months and 1577 clutches) and 4 years from Maputaland (2009–2012; n = 8 months and 31 clutches).

We used hatching success (proportion of hatched eggs in a clutch) and emergence rate (proportion of hatchlings in a nest that emerge in relation to the number of hatchlings that hatched) as estimations of hatchling output. At each site we estimated hatching success (*H*) and emergence rate (*R*) using the formulas *H* = *S* / (*S* + *U*) and *R* = (*S* – (*L* + *D*))/*S* respectively, where *S* is number of hatched shells, *U* number of unhatched eggs, *L* number of hatchlings found alive in the nest and *D* number of hatchlings found dead in the nest excavation^27^. We estimated monthly mean hatching success and emergence rate (±SD) by month eggs were laid at each site and assessed the effects of local climatic conditions. We excluded nests that were eroded, predated or poached from the analyses.

Local climate data were obtained from weather stations located at nearby airports. We used data from the Daniel Oduber International airport (~45 km from Playa Grande), Limón/Cienaguita airport (~38 km from Pacuare), Henry E. Rohlsen Airport (~10 km from Sandy Point) and Mbazwana airfield (~10 km from the nesting beach in Maputaland). For each location, we obtained mean air temperatures and precipitation accumulated per month. Climate data were obtained from the National Meteorological Institute of Costa Rica for Playa Grande and Pacuare, from the National Oceanic and Atmospheric Administration of the United States (NOAA, http://www.ncdc.noaa.gov/cdo-web/datasets) for Sandy Point and from the South African Weather Service for Maputaland.

We used air temperature and precipitation outputs from three global climate models that were assessed to resolve present-day ENSO dynamics and feedbacks^40^. These climate models were CSIRO MK6, GFDL CM3 and INM CM4 and earlier versions of these models were used for the Playa Grande nesting site in previous studies^4^,^16^. We used deltas between the high concentration scenario representative concentration pathway (RCP) 8.5 and historical runs from the Intergovernmental Panel on Climate Change (IPCC) fifth assessment to obtain climatic projections of air temperature and precipitation for each nesting site throughout the 21^st^ century. We bias-corrected global climate model output (both mean and variability) based on local observations from each weather station following previous methodology^4^,^16^.

### Data analyses

We used a One-way ANOVA to compare hatching success and emergence rate between sites. We used the Tukey test for hatching success comparisons because data met assumption of homogeneity of variances and Tamhane’s test for comparisons of the emergence rate among sites because variances were not homogenous.

We used Generalized Additive Models (GAM) to test the non-linear relationship between local climate and hatching success and emergence rate. The additive model estimates a non-parametric function for each predictor and has the benefit of avoiding prior assumptions about the shape of the function^41^. Smoothing splines produce a smooth generalization of the relationship between the two variables in a plot for visual examination. Predictor variables included in the models were: air temperature during the two months of incubation (Temp), average rain accumulated during incubation months (Rain_ave_), total rain accumulated during incubation (Rain_acc(0,1)_), rain accumulated during (a) the month eggs were laid (Rain_acc(0)_), (b) the month eggs were laid and previous month (Rain_acc(0,-1)_), (c) the month eggs were laid and two previous months (Rain_acc(0,-1,-2)_) and (d) the two months before eggs were laid (Rain_acc(-1,-2)_). Predictor variables used in each model are detailed in supplementary Tables S1 and S2. We used the Corrected Akaike Information Criterion (AICc) to select the best model for each nesting site. Due to the lower sample size available from Maputaland (4 seasons, 8 months), models with 9 or greater maximum degrees of freedom had fewer unique covariate combinations than specified maximum degrees of freedom and could not run the analyses. Therefore, we reduced the maximum degrees of freedom to 8 df. We used the mgcv library in R, version 3.0.1 for GAMs and used SPSS statistics v. 20.0 for other statistical analyses.

## Additional Information

**How to cite this article**: Santidrián Tomillo, P. *et al.* Global analysis of the effect of local climate on the hatchling output of leatherback turtles. *Sci. Rep.* **5**, 16789; doi: 10.1038/srep16789 (2015).

## Supplementary Material

Supplementary Information

## Figures and Tables

**Table 1:**
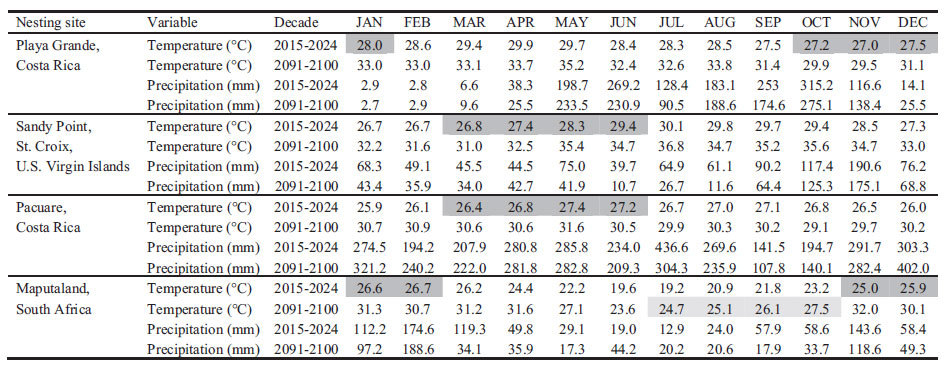
Projections of air temperature (°C) and precipitation (mm) throughout the 21^st^ century for nesting sites used by leatherback turtles around the world.

Locations were in the eastern Pacific (Playa Grande, Costa Rica), Caribbean Sea (Sandy Point, St. Croix and Pacuare, Costa Rica) and Indian Ocean (Maputaland, South Africa). Dark gray area shows months in the nesting season. Light gray area shows months that are projected to have temperatures similar to current nesting season conditions by the end of the 21^st^ century.

**Table 2:** Leatherback turtle nesting sites included in the study, annual number of nesting turtles and nests, monitoring area (beach length), apparent population trends, classification by the International Union for Conservation of Nature (IUCN) and main anthropogenic threats to each nesting population.

Nesting site	Ocean basin	Beach length (km)	Annual number of nesting turtles	Annual number of nests	Trends	IUCN assessment	Anthropogenic threats
Playa Grande, Costa Rica	Eastern Pacific	3.6	~30–40^10^*	~165–265^10^	declining	critically endangered	fisheries, egg poaching, tourist development
Sandy Point, US Virgin Islands	Western Atlantic	3	~200^42^	—	increasing	least concern	fisheries
Pacuare, Costa Rica	Western Atlantic	5.7	~300–500^43^	~500–1200^43^	stable	least concern	fisheries, egg poaching
Maputaland, South Africa	Indian	53	~70^44^	~200–400^44^	stable or declining	critically endangered	fisheries

^*^estimation includes years between 2008 and 2013 to account for declining trend.
