# Supplementary material for: Global analysis of the effect of local climate on the hatchling output of leatherback turtles
Source: Sci Rep. 2015 Nov 17;5:16789. doi: 10.1038/srep16789 (PMC4648107; doi:10.1038/srep16789)

Global analysis of the effect of local climate on the hatchling output of leatherback turtles

Pilar Santidrián Tomillo[1*], Vincent S. Saba[2], Claudia D. Lombard[3], Jennifer M. Valiulis[4], Nathan J. Robinson[5], Frank V. Paladino[5], James R. Spotila[6], Carlos Fernández[7], Marga L. Rivas[8], Jenny Tucek[9], Ronel Nel[9] & Daniel Oro[1]

[1]Population Ecology Group, Institut Mediterrani d' Estudis Avançats, IMEDEA (CSIC-UIB), Miquel Marquès, 21, 07190, Esporles, Mallorca, Spain.

[2]National Oceanic and Atmospheric Administration, National Marine Fisheries Service, Northeast Fisheries Science Center, c/o Geophysical Fluid Dynamics Laboratory, 201 Forrestal Road, Princeton University Forrestal Campus, Princeton, New Jersey, USA;

[3]U.S. Fishing and Wildlife Service, USA

[4]Geographic Consulting, St. Croix, U.S. Virgin Islands, USA.

[5]Department of Biology, Indiana-Purdue University, Fort Wayne, Indiana, USA.

[6]Department of Biodiversity, Earth and Environmental Science, Drexel University, Philadelphia, Pennsylvania, USA.

[7]Endangered Wildlife Trust, Avda. 11, San José, Costa Rica.

[8]Universidad de Granada, Campus Fuentenueva s/n, Granada, Spain.

[9]Department of Zoology, Nelson Mandela Metropolitan University, Port Elizabeth, South Africa.

Table S1 | Generalized Additive Model (GAM) results on the effect of local climate on hatching success of leatherback turtle clutches. Predictors were air temperature (Temp), average rain accumulated during incubation ($Rain_{ave}$), total rain accumulated during incubation ($Rain_{acc(0,1)}$), rain accumulated during the month eggs were laid ($Rain_{acc(0)}$), rain accumulated during the month eggs were laid and previous month ($Rain_{acc(0,-1)}$), rain accumulated during the month eggs were laid and two previous months ($Rain_{acc(0,-1,-2)}$) and rain accumulated during two months before eggs were laid ($Rain_{acc(-1,-2)}$). Best models (lowest AICc) are marked in bold and significant effect from predictor with (*). Only sites for which there were significant effects of local climate are included.

**Model (smooth terms)**	**Site**	**df**	**AIC**	**AICc**	**Deviance explained (%)**	**R^2 adjusted**
**Temp+Rain*$_{ave(0,1)}$**	**All**	**11.4**	**-231.0**	**-229.1**	**33.8**	**0.296**
**Temp+Rain*$_{acc(0,1)}$**	**All**	**11.4**	**-231.0**	**-229.1**	**33.8**	**0.296**
Temp+Rain*$_{acc(0)}$	All	10.8	-209.9	-208.2	23.9	0.195
Temp+Rain*$_{acc(0,-1)}$	All	11.7	-210.3	-208.4	25.0	0.201
Temp+Rain*$_{acc(0,-1,-2)}$	All	8.7	-190.8	-189.7	12.0	0.082
Temp+$Rain_{acc(-1,-2)}$	All	8.7	-187.6	-186.5	10.3	0.064
Temp	All	3.9	-185.3	-185.0	3.5	0.023
Rain*$_{ave}$	All	10.0	-228.5	-227.0	31.5	0.279
Rain*$_{acc(0,1)}$	All	10.0	-228.5	-227.0	31.5	0.279
Rain*$_{acc(0)}$	All	9.0	-207.9	-206.7	21.3	0.176
Rain*$_{acc(0,-1)}$	All	9.9	-209.0	-207.6	22.7	0.187
$Rain_{acc(0,-1,-2)}$	All	7.2	-189.4	-188.6	9.7	0.067

$Rain_{acc(-1,-2)}$	All	7.1	-186.0	-185.3	7.6	0.046
$Temp+Rain*_{ave(0,1)}$	S Point	6.4	-157.1	-155.9	12.7	0.081
$Temp+Rain*_{acc(0,1)}$	S Point	6.4	-157.1	-155.9	12.7	0.081
$Temp+Rain*_{acc(0)}$	S Point	6.7	-155.8	-154.5	11.9	0.071
$Temp+Rain*_{acc(0,-1)}$	S Point	7.3	-160.5	-159.0	17.6	0.124
**$Temp+Rain*_{acc(0,-1,-2)}$**	**S Point**	**8.2**	**-165.4**	**-163.5**	**23.6**	**0.178**
$Temp+Rain*_{acc(-1,-2)}$	S Point	8.5	-158.5	-156.5	18	0.115
Temp	S Point	3.0	-152.3	-152.1	0.75	-0.003
$Rain*_{ave}$	S Point	3.0	-158.5	-158.2	7.29	0.006
$Rain*_{acc(0,1)}$	S Point	3.0	-158.5	-158.2	7.29	0.006
$Rain*_{acc(0)}$	S Point	3.0	-157.0	-156.7	5.71	0.046
$Rain*_{acc(0,-1)}$	S Point	3.0	-158.9	-158.6	7.73	0.067
$Rain*_{acc(0,-1,-2)}$	S Point	3.4	-161.8	-161.5	11.6	0.101
$Rain_{acc(-1,-2)}$	S Point	3.7	-156.6	-156.2	6.78	0.050
$Temp+Rain_{ave(0,1)}$	P Grande	7.2	-44.7	-40.5	47.4	0.383
$Temp+Rain_{acc(0,1)}$	P Grande	7.2	-44.7	-40.5	47.4	0.383
$Temp+Rain_{acc(0)}$	P Grande	7.8	-44.0	-39.0	48.2	0.379
$Temp+Rain*_{acc(0,-1)}$	P Grande	5.4	-48.9	-46.5	48.5	0.429
$Temp+Rain*_{acc(0,-1,-2)}$	P Grande	5.4	-60.4	-58	62.4	0.584
$Temp+Rain*_{acc(-1,-2)}$	P Grande	5.4	-63.3	-61.1	65.3	0.617
Temp*	P Grande	4.8	-43.1	-41.2	37.1	0.317
$Rain*_{ave}$	P Grande	5.6	-40.5	-38.0	35.6	0.282
$Rain*_{acc(0,1)}$	P Grande	5.6	-40.5	-38.0	35.6	0.282

$Rain^*_{acc(0)}$	P Grande	6.9	-41.7	-37.8	41.7	0.324
$Rain^*_{acc(0,-1)}$	P Grande	3.9	-46.7	-45.5	40.2	0.368
$Rain^*_{acc(0,-1,-2)}$	P Grande	3.9	-61.2	-60.0	60	0.578
**$Rain^*_{acc(-1,-2)}$**	**P Grande**	**3.5**	**-63.5**	**-62.5**	**61.8**	**0.60**
$Temp+Rain_{ave(0,1)}$	Pacuare	4.0	-40.5	-38.7	6.76	-0.01
$Temp+Rain_{acc(0,1)}$	Pacuare	4.0	-40.5	-38.7	6.76	-0.01
$Temp+Rain_{acc(0)}$	Pacuare	4.0	-40.5	-38.7	6.7	-0.01
$Temp+Rain_{acc(0,-1)}$	Pacuare	6.5	-44.8	-39.7	34	0.201
$Temp+Rain_{acc(0,-1,-2)}$	Pacuare	7.0	-41.4	-35.5	27.9	0.107
$Temp+Rain_{acc(-1,-2)}$	Pacuare	8.6	-44.0	-34.4	42	0.221
Temp	Pacuare	3.0	-42.3	-41.2	5.88	0.021
$Rain_{ave}$	Pacuare	3.0	-41.3	-40.3	2.65	-0.012
$Rain_{acc(0,1)}$	Pacuare	3.0	-41.3	-40.3	2.65	-0.012
$Rain_{acc(0)}$	Pacuare	3.0	-41.2	-40.1	2.07	-0.018
**$Rain_{acc(0,-1)}$**	**Pacuare**	**5.5**	**-45.8**	**-42.3**	**31.6**	**0.209**
$Rain_{acc(0,-1,-2)}$	Pacuare	7.0	-43.7	-37.8	33.7	0.179
$Rain^*_{acc(-1,-2)}$	Pacuare	10.2	-54.2	-39.7	64.6	0.483

Table S2 | Generalized Additive Model (GAM) results on the effect of local climate on emergence rate of leatherback turtle clutches. Predictors were air temperature (Temp), average rain accumulated during incubation ($Rain_{ave}$), total rain accumulated during incubation ($Rain_{acc(0,1)}$), rain accumulated during the month eggs were laid ($Rain_{acc(0)}$), rain accumulated during the month eggs were laid and previous month ($Rain_{acc(0,-1)}$), rain accumulated during the month eggs were laid and two previous months ($Rain_{acc(0,-1,-2)}$) and rain accumulated during two months before eggs were laid ($Rain_{acc(-1,-2)}$). Best models (lowest AICc) are marked in bold and effect from significant predictor with (*). Only sites for which there were significant effects of local climate are included.

**Model (smooth terms)**	**Site**	**df**	**AIC**	**AICc**	**Deviance explained (%)**	**R^2 adjusted**
Temp+$Rain*_{ave(0,1)}$	All	11.0	-428.2	-426.4	36.1	0.323
Temp+$Rain*_{acc(0,1)}$	All	11.0	-428.2	-426.4	36.1	0.323
Temp+$Rain*_{acc(0)}$	All	11.3	-402.8	-401.0	25.5	0.209
Temp+$Rain*_{acc(0,-1)}$	All	12.1	-406.9	-404.7	28.1	0.233
Temp+$Rain_{acc(0,-1,-2)}$	All	6.5	-374.4	-373.7	5.68	0.029
Temp+$Rain*_{acc(-1,-2)}$	All	10.9	-384.2	-382.4	16	0.110
Temp	All	4.0	-375.3	-375.1	3.24	0.020
**$Rain*_{ave}$**	**All**	**10.0**	**-430.1**	**-428.7**	**36.1**	**0.327**
**$Rain*_{acc(0,1)}$**	**All**	**10.0**	**-430.1**	**-428.6**	**36.1**	**0.327**
$Rain*_{acc(0)}$	All	9.5	-402.6	-401.3	23.7	0.20
$Rain*_{acc(0,-1)}$	All	10.3	-407.1	-405.5	26.6	0.225

$Rain_{acc(0,-1,-2)}$	All	6.0	-375.4	-374.9	5.64	0.032
$Rain*_{acc(-1,-2)}$	All	9.1	-385.2	-384.0	14.6	0.106
$Temp+Rain_{ave(0,1)}$	S Point	4.0	-383.5	-383.0	1.67	-0.005
$Temp+Rain_{acc(0,1)}$	S Point	4.0	-383.5	-383.0	1.67	-0.005
$Temp+Rain_{acc(0)}$	S Point	4.0	-382.9	-382.4	0.95	-0.013
$Temp+Rain*_{acc(0,-1)}$	S Point	7.9	-385.5	-383.8	11.9	0.056
$Temp+Rain*_{acc(0,-1,-2)}$	S Point	7.6	-387.7	-386.1	13.3	0.075
$Temp+Rain*_{acc(-1,-2)}$	S Point	4.2	-389.5	-389.0	8.46	0.061
Temp	S Point	3.0	-384.3	-384.0	0.31	-0.008
$Rain_{ave}$	S Point	3.0	-385.3	-385.0	1.46	0.003
$Rain_{acc(0,1)}$	S Point	3.0	-385.3	-385.0	1.46	0.003
$Rain_{acc(0)}$	S Point	3.0	-384.6	-384.3	0.69	-0.004
$Rain*_{acc(0,-1)}$	S Point	3.0	-388.5	-388.2	4.84	0.038
$Rain*_{acc(0,-1,-2)}$	S Point	3.0	-390.8	-390.5	7.26	0.062
**$Rain*_{acc(-1,-2)}$**	**S Point**	**3.2**	**-391.5**	**-391.2**	**8.4**	**0.072**
$Temp+Rain*_{ave(0,1)}$	P Grande	8.4	-80.5	-74.6	55.7	0.458
$Temp+Rain*_{acc(0,1)}$	P Grande	8.4	-80.5	-74.6	55.7	0.458
$Temp+Rain*_{acc(0)}$	P Grande	7.7	-81.4	-76.6	55	0.463
$Temp+Rain*_{acc(0,-1)}$	P Grande	6.0	-84.5	-81.6	54.7	0.489
$Temp*+Rain*_{acc(0,-1,-2)}$	P Grande	12.7	-96.5	-80.7	77.7	0.678
$Temp+Rain*_{acc(-1,-2)}$	P Grande	8.7	-84.6	-78.2	61	0.518
Temp*	P Grande	5.5	-73.6	-71.2	36.9	0.30
$Rain*_{ave}$	P Grande	4.9	-77.4	-75.5	41.4	0.361

Rain*$_{acc(0,1)}$	P Grande	4.9	-77.4	-75.5	41.4	0.361
Rain*$_{acc(0)}$	P Grande	4.8	-78.1	-76.3	42.1	0.371
Rain*$_{acc(0,-1)}$	P Grande	4.5	-85.6	-84.0	52.2	0.485
**Rain*$_{acc(0,-1,-2)}$**	**P Grande**	**4.2**	**-89.0**	**-87.5**	**55.8**	**0.528**
Rain*$_{acc(-1,-2)}$	P Grande	3.8	-79.9	-78.7	42	0.387
Temp+Rain$_{ave(0,1)}$	Pacuare	4.0	-46.0	-44.2	0.8	-0.075
Temp+Rain$_{acc(0,1)}$	Pacuare	4.0	-46.0	-44.2	0.8	-0.075
Temp+Rain$_{acc(0)}$	Pacuare	4.0	-45.9	-44.1	0.2	-0.082
Temp+Rain$_{acc(0,-1)}$	Pacuare	7.3	-53.7	-47.3	41.3	0.264
Temp+Rain$_{acc(0,-1,-2)}$	Pacuare	9.6	-48.0	-35.7	38.9	0.138
Temp+Rain*$_{acc(-1,-2)}$	Pacuare	10.8	-61.7	-44.8	66.5	0.492
Temp	Pacuare	3.0	-47.8	-46.8	0.0	-0.039
Rain$_{ave}$	Pacuare	3.0	-48.0	-47.0	0.7	-0.032
Rain$_{acc(0,1)}$	Pacuare	3.0	-48.0	-47.0	0.7	-0.032
Rain$_{acc(0)}$	Pacuare	3.0	-47.9	-46.8	0.1	-0.039
**Rain$_{acc(0,-1)}$**	**Pacuare**	**6.2**	**-55.2**	**-50.7**	**40.0**	**0.284**
Rain$_{acc(0,-1,-2)}$	Pacuare	3.0	-48.1	-47.0	0.9	-0.031
Rain*$_{acc(-1,-2)}$	Pacuare	9.9	-62.7	-49.4	65.3	0.503

Figure S1 | Effect of precipitation on the hatchling output. Values represent mean monthly hatching success and emergence rate. Best predictors on hatching success were precipitation accumulated during **(a)** the month eggs were laid and the two previous months at Sandy Point, **(b)** the month eggs were laid and the month before at Pacuare and **(c)** the two months before eggs were laid at Playa Grande (R^2 adjusted in GAMs = 0.178, 0.209 and 0.60 for Sandy Point, Pacuare and Playa Grande respectively, table S1). Emergence rate predictors were precipitation accumulated **(d)** two months before eggs were laid at Sandy Point, **(e)** during the month eggs were laid and the month before at Pacuare and **(f)** in the month eggs were laid and two months before at Playa Grande (R^2 adjusted in GAMs = 0.072, 0.284 and 0.528 for Sandy Point, Pacuare and Playa Grande respectively, table S2).

Fig. S1.

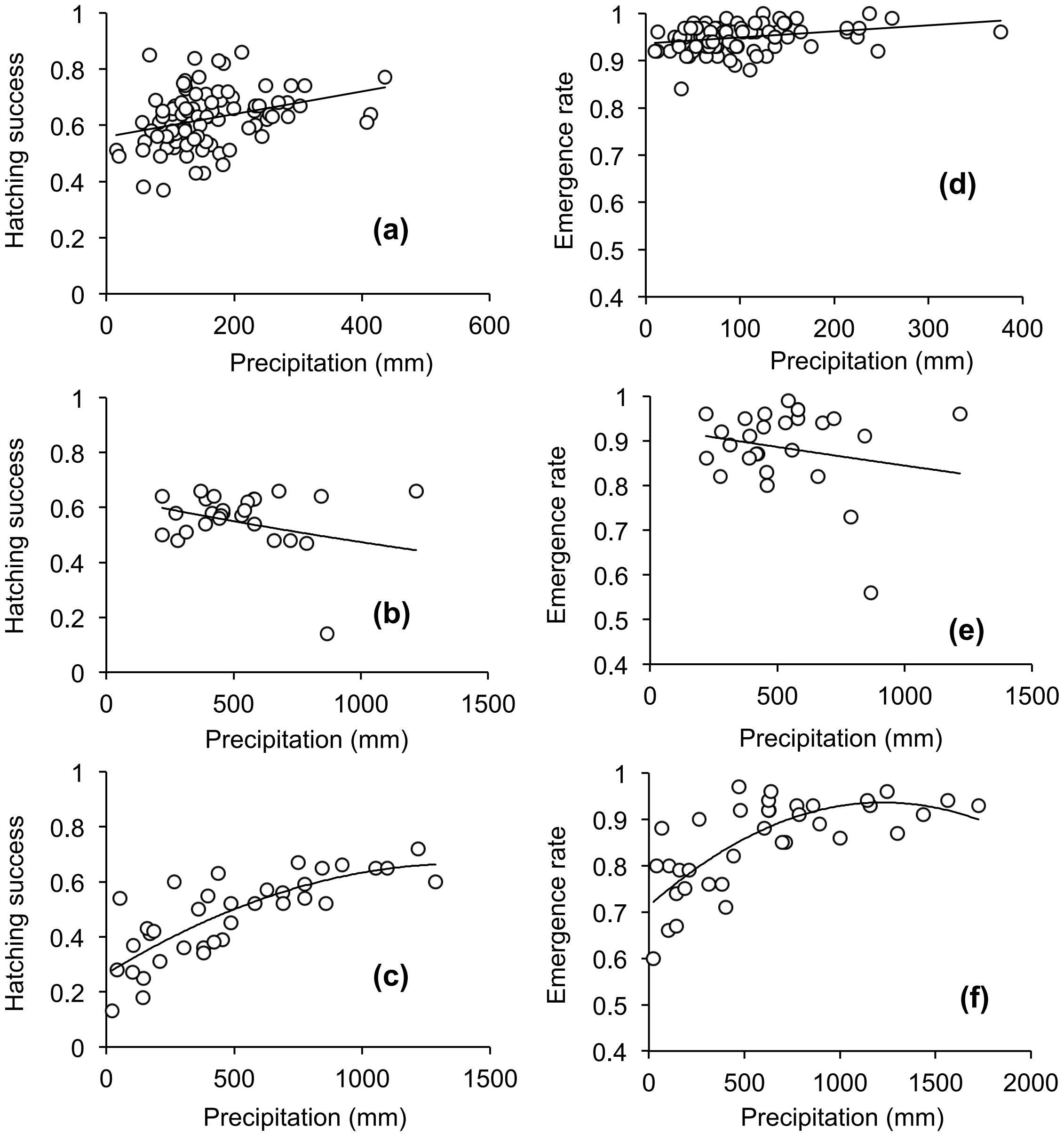

Supplement: Supplementary Information [file srep16789-s1.pdf]
